# Diprotonation of taurine: 2-[dihy­droxy(oxo)sul­fan­ylium­yl]ethanaminium bis[hexa­fluoro­arsenate(V)]

**DOI:** 10.1107/S2053229624010489

**Published:** 2024-11-20

**Authors:** Valentin Bockmair, Andreas Klöck, Dirk Hollenwäger, Andreas J. Kornath

**Affiliations:** a Department Chemie, Ludwig Maximilian University of Munich, Butenandtstrasse 5-13 (Haus D), D-81377 München, Germany; University of Sydney, Australia

**Keywords:** taurine, superacid, amino­alkyl­sulfonic acid, crystal structure, protonation, vibrational spectroscopy, NMR spectroscopy

## Abstract

Taurine is part of the cysteine cycle and is one of the few naturally occuring organosulfur-based mol­ecules in the human body. As implied by modern studies, protonated taurine is of biological impact. The first attempts to isolate its protonated species in the binary superacidic system HF/SbF_5_ were performed by Hopfinger, resulting in the isolation of monoprotonated taurine. Since the chosen conditions seemed rather harsh, investigations in less acidic systems were performed at room tem­per­a­ture to explore the involved protonated species. Herein, we present the structure of 2-[dihy­droxy(oxo)sulfanylium­yl]ethanaminium bis[hexa­fluorido­arsenate(V)], [H_2_O_3_SC_2_H_4_NH_3_][AsF_6_]_2_, the diprotonated form of 2-amino­ethane­sulfonic acid (taurine). It was synthesized in the binary superacidic system HF/AsF_5_ and crystallizes as colourless needles. Diprotonated taurine was structurally characterized by single-crystal X-ray diffraction analysis, low-tem­per­a­ture vibrational spectroscopy and NMR spectroscopy.

## Abstract 2

Taurine is one of the most common natural substances, despite its behaviour under stongly acidic condtions, which was first uncovered with the determination of the crystal structure of 2-sulfo­ethyl­ammonium hexa­fluorido­anti­monate. We report further protonation, forming the sul­fo­nium ion, resulting from a tem­per­a­ture-dependent reaction of taurine with the superacidic system HF/AsF_5_.

## Introduction

Taurine represents the smallest naturally occuring amino­alkyl­sulfonic acid. It was discovered in 1827 by Gmelin and Tiedemann, and its crystal structure was investigated in 1963 for the first time (Sutherland & Young, 1963 ▸). Its existence in the form of a zwitterion was proven by Okaya (1966 ▸). Being a part of the cysteine metabolism cycle, taurine appears naturally in animal and human bodies as a product of enzymatic catalysis *via* oxidation, deca­rboxylation and further oxidation under energy consumption. Depending on physiology, it is mainly stored in muscle cells. New studies have revealed the biological impact of protonated taurine by inhibition of connexin 26-containing channels (Tao & Harris, 2004 ▸). By spatial separation, strong acidic conditions can prevail in com­partments, which might lead to the protonation of taurine. Limited by the levelling effect, it might only exist as a short-lived species in enzymatic catalysis in such com­partments.

The first protonated structure of taurine was reported for 2-sulfo­ethyl­ammonium hexa­fluorido­anti­monate(V), [HO_3_SC_2_H_4_NH_3_][SbF_6_], together with its spectroscopic data (Hop­finger *et al.*, 2011 ▸; Hopfinger, 2012 ▸). Since this first protonation of taurine, which was achieved at −50 °C, the question arises whether physiological conditions, especially room tem­per­a­ture, could lead to higher states of protonation. Although sulfonic acid moieties (HO_3_S–*R*) are known to be strong acids, for example, chloro­sulfonic acid, which already belongs to the class superacids, they can be protonated with the formation of their corresponding sul­fo­nium cations. Furthermore, diprotonation of sulfonic acid moieties ([H_3_O_3_S–*R*]^2+^) has not been observed so far.

Regarding other known structures containing sul­fo­nium moieties ([H_2_O_3_S–*R*]^+^) with more acidic side chains (Soltner *et al.*, 2011 ▸; Seelbinder *et al.*, 2010 ▸), it is also possible that a weaker acidic system might succeed in protonating taurine or even 2-sulfo­ethyl­ammonium. Therefore, we investigated the protonation of taurine in the binary superacidic system HF/AsF_5_ at room tem­per­a­ture.

## Experimental

**Caution!** Note that any contact with the described com­pounds should be avoided. Hydrolysis of AsF_5_ and the synthesized salts forms HF which burns skin and causes irreparable damage. Safety precautions should be taken while handling these com­pounds. All reactions were carried out by employing standard Schlenk techniques on a stainless steel vacuum line. The syntheses of the salts were performed using FEP/PFA reactors with stainless steel valves.

### Synthesis and crystallization

Anhydrous hy­dro­gen fluoride (80.04 mg, 4.0 mmol) and arsenic penta­fluoride (339.82 mg, 2.0 mmol) were condensed into a FEP reactor under liquid nitrogen cooling. The solution was warmed to −78 °C and thoroughly mixed for 5 min. Taurine (125.14 mg, 1.0 mmol) was added to the superacid after freezing it at liquid nitro­gen tem­per­a­ture and the solution was warmed to room tem­per­a­ture again and thoroughly mixed for 5 min. The volatile com­ponents were removed over 12 h *in vacuo* at −78 °C. The product, [H_2_O_3_SC_2_H_4_NH_3_][AsF_6_]_2_, (I) (Scheme 1), was ob­tained in the form of colourless needles in qu­anti­tative yield.
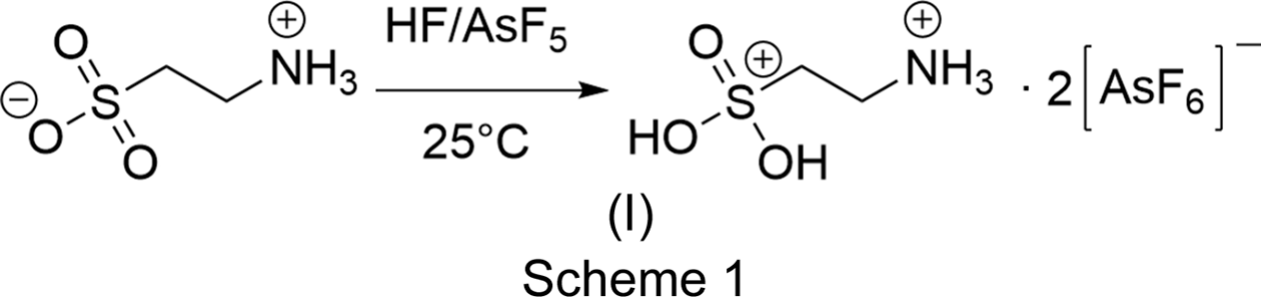


### Crystal structure refinement

Basic crystallographic data, details on data collection, and structure refinement are summarized in Table 1 ▸. The positions of the H atoms in the structure were localized in a difference Fourier map and refined without any restrictions. All atoms occupy the general position 4*a* since no special positions exist in *P*2_1_2_1_2_1_.

### Analysis

The product, (I), was characterized by single-crystal X-ray diffraction, low-tem­per­a­ture vibrational spectroscopy and NMR spectroscopy.

Low-tem­per­a­ture vibrational spectroscopy measurements were performed to confirm the conformation and protonation state of taurine. IR spectroscopic investigations were carried out with a Bruker VERTEX 80V FT–IR spectrometer using a cooled cell with a single-crystal CsBr plate on which small amounts of the sample were placed (Bayersdorfer *et al.*, 1972 ▸). For Raman measurements, a Bruker MultiRam FT–Raman spectrometer with Nd:YAG laser excitation (λ = 1064 nm) was used. The measurement was performed after transferring the sample into a cooled (−196 °C) glass cell under a nitro­gen atmosphere and subsequent evacuation of the glass cell. The low-tem­per­a­ture spectra are depicted in the supporting in­for­mation (Fig. S1).

Single crystals of [H_2_O_3_SC_2_H_4_NH_3_][AsF_6_]_2_, (I), suitable for single-crystal diffraction analysis were selected under a stereo microscope in a cooled nitro­gen stream. The single crystal was prepared on a stainless steel polyamide micromount and data collection was performed at 112 K on an Xcalibur diffrac­tom­eter system (Rigaku Oxford Diffraction). For the diffraction pattern of (I) and an image of the single crystal on the polyamide loop of the micromount, see Fig. S2 of the sup­porting information.

NMR measurements were performed on a Bruker AV400 TR spectrometer at various tem­per­a­tures. NMR samples were measured in FEP tubes inlaid with anhydrous HF as solvent, and acetone-*d*_6_ was used as an external reference. The NMR spectra are depicted in the supporting information (Figs. S3–S13).

### Quantum chemical calculations

Quantum chemical calculation were based on the single-crystal structure of diprotonated taurine using the DFT (B3LYP) and MP2 methods at the cc-pVTZ-aug level of theory with *Gaussview*/*GAUSSIAN16* software (Dennington *et al.*, 2016 ▸; Frisch *et al.*, 2016 ▸).

**Figure 1:**
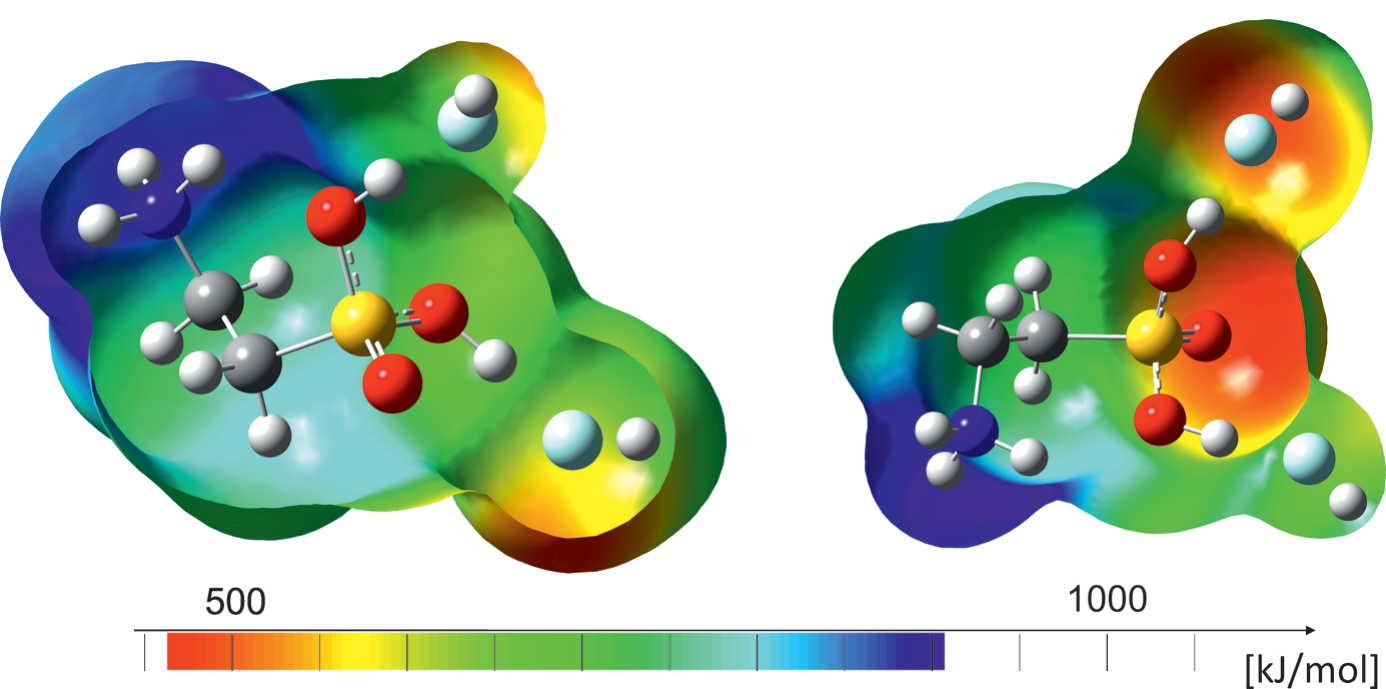
Calculated electrostatic potential mapped onto an electron-density isosurface value of 0.0004 bohr^−3^ with the colour scale ranging from 464.714 (red) to 905.798 kJ mol^−1^ (blue) of [H_2_O_3_SC_2_H_4_NH_3_][AsF_6_]_2_ for two different orientations.

For calculations, the hy­dro­gen bonds were simulated by adding two additional HF mol­ecules to the cation in the gas phase (Fig. 1 ▸), resulting in more suitable calculated vibrational frequencies, in particular with regard to the vibrations of the hy­droxy groups. This method is already established in the literature in order to simulate gas phase basicity (Soltner *et al.*, 2011 ▸).

The structure of the cation and simulated contacts was optimized with DFT methods and vibration frequencies were calculated as reported in Table 2 ▸. For the calculation of more accurate energy values of the mapped electrostatic potential, MP2 methods were used based on the optimized structure.

As visualized by the mapped electrostatic potential of diprotonated taurine, the positive charge on the S atom is partially shifted along the carbon backbone. The positive potential of the ammonium group represents the maximum of the positive potential (blue), which is in good agreement with its cationic state. The minimum of the positive potential is located on the sulfuryl O atom (red).

## Results and discussion

### Vibrational spectroscopy

The observed experimental vibration frequencies for diprotonated taurine were assigned to the anion and cation (Tables 2 ▸ and 3 ▸) in accordance with quantum chemical calculations (DFT-B3LYP/aug-cc-pVTZ) and com­pared to the vibrational spectroscopic data for the monoprotonated species (Hopfinger, 2012 ▸).

*C*_1_ symmetry was assigned to the diprotonated species of taurine with 42 fundamental vibrations, which are com­piled in Table 2 ▸.

While the vibrations along the ethyl­ammonium chain only differ weakly com­pared to monoprotonation, the vibrations of the sul­fo­nium moiety show a split of the SO_3_ vibrations and coupled C—S stretching vibrations.

For the [AsF_6_]^−^ anions, more vibrations were observed than expected, due to solid-state effects leading to a lowered symmetry com­pared to an ideal octa­hedral coordination (Table 3 ▸).

### Crystal structure

**Figure 2:**
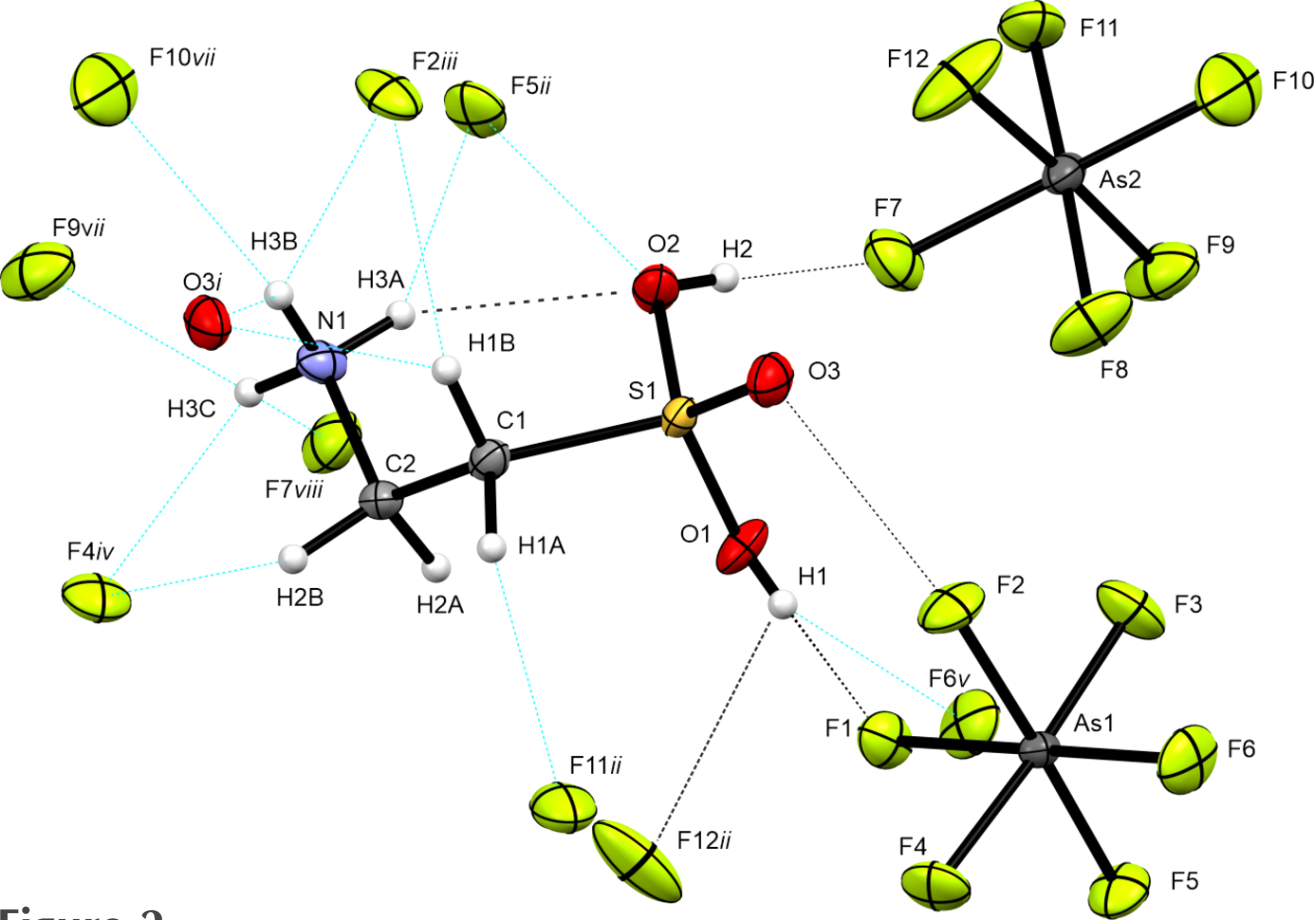
The asymmetric unit of [H_2_O_3_SC_2_H_4_NH_3_][AsF_6_]_2_. Displacement ellipsoids are drawn at the 50% probability level.

**Figure 3:**
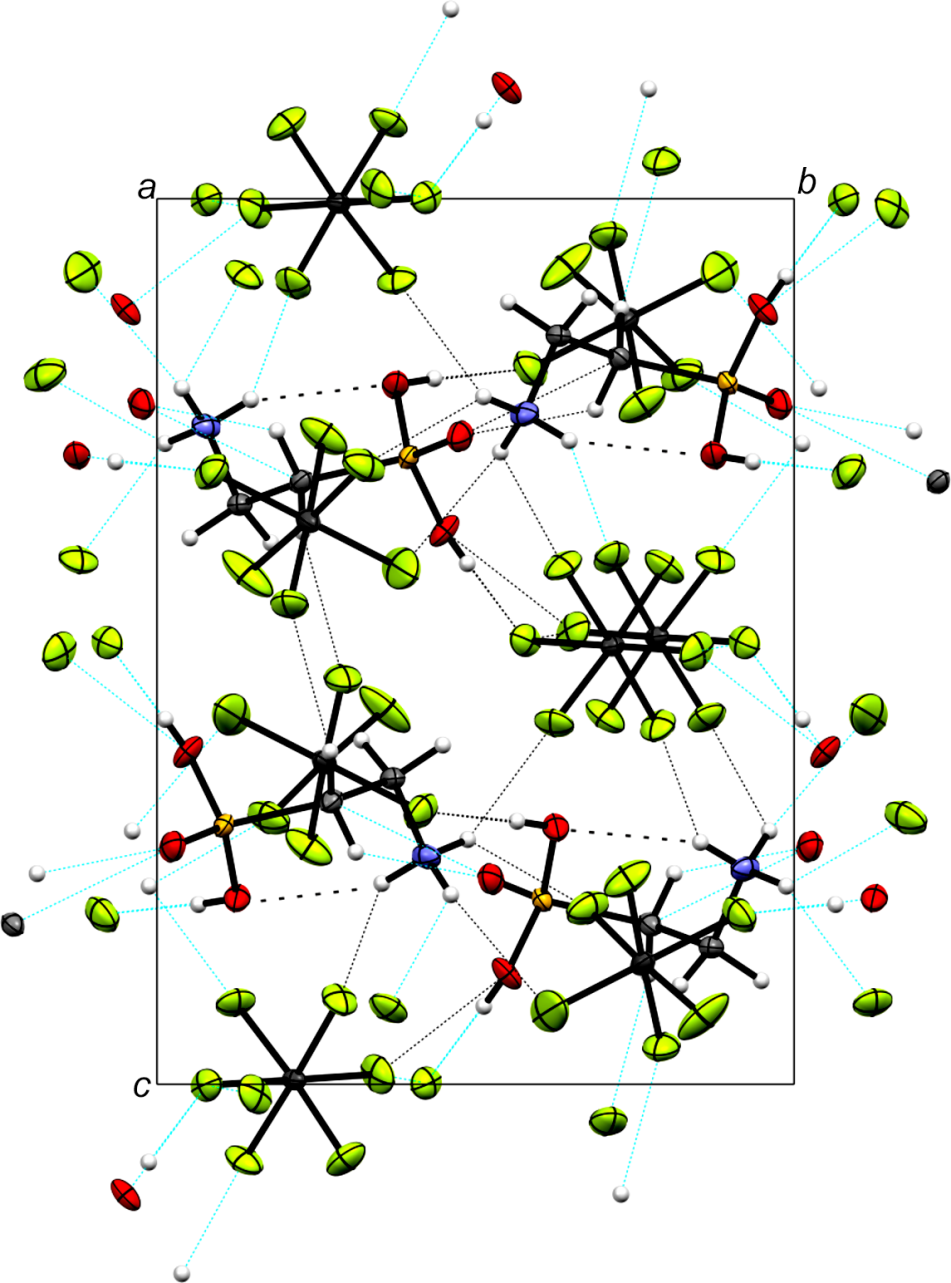
The crystal structure of [H_2_O_3_SC_2_H_4_NH_3_][AsF_6_]_2_, viewed along the *a* axis. Displacement ellipsoids are drawn at the 50% probability level.

As implied by the two [AsF_6_]^−^ anions in the asymmetric unit (Fig. 2 ▸), taurine forms a dication with protonation to the sulfonate moiety. The crystal structure of diprotonated taurine (Fig. 3 ▸) is built up on a three-dimensonal network of many inter­actions, especially hy­dro­gen bonds (Table 4 ▸).

In the diprotonated taurine species, the S1—C1—C2—N1 torsion angle of −79.7 (5)° is enlarged [Δ(torsion) = 6.2°] com­pared to the monoprotonated species, allowing more inter­actions with the isolated [AsF_6_]^−^ anions. This conformation leads to a weakening of the intra­molecular hy­dro­gen bond to 2.998 Å [Δ(N—H⋯O) = 0.091 Å], but increases the number of fluorine acceptors for the inter­molecular hy­dro­gen bonds.

The sul­fo­nium moiety shows two S—O bonds of similar length for the hy­droxy O atoms [S1—O1 = 1.511 (4) Å and S1—O2 = 1.513 (4) Å], which appear to be slightly shortened in com­parison with the monoprotonated species [Δ(S—O) = −0.036 Å]. The bond length in the sulfuryl group is significantly shortened [S1—O3 = 1.410 (4) Å] com­pared with monoprotonation [1.437 (2) Å] [Δ(S=O) = −0.027 Å]. The bond lengths of the sul­fo­nium moiety are nearly equal to the values reported by Soltner for the tri­fluoro­methane­sul­fo­nium ion (Soltner *et al.*, 2011 ▸).

The C1—S1, C1—C2 and C1—N1 bond lengths differ only marginally considering the influence of protonation, which can be justified by the orbital situation as the S atom does not participate in hyperconjugation along the ammonium­alkyl chain. Therefore, substituent effects on the chain cause larger changes of the bond lengths in the chain (Soltner *et al.*, 2011 ▸). For com­parison with calculated data and related structures, see Table 5 ▸.

**Figure 4:**
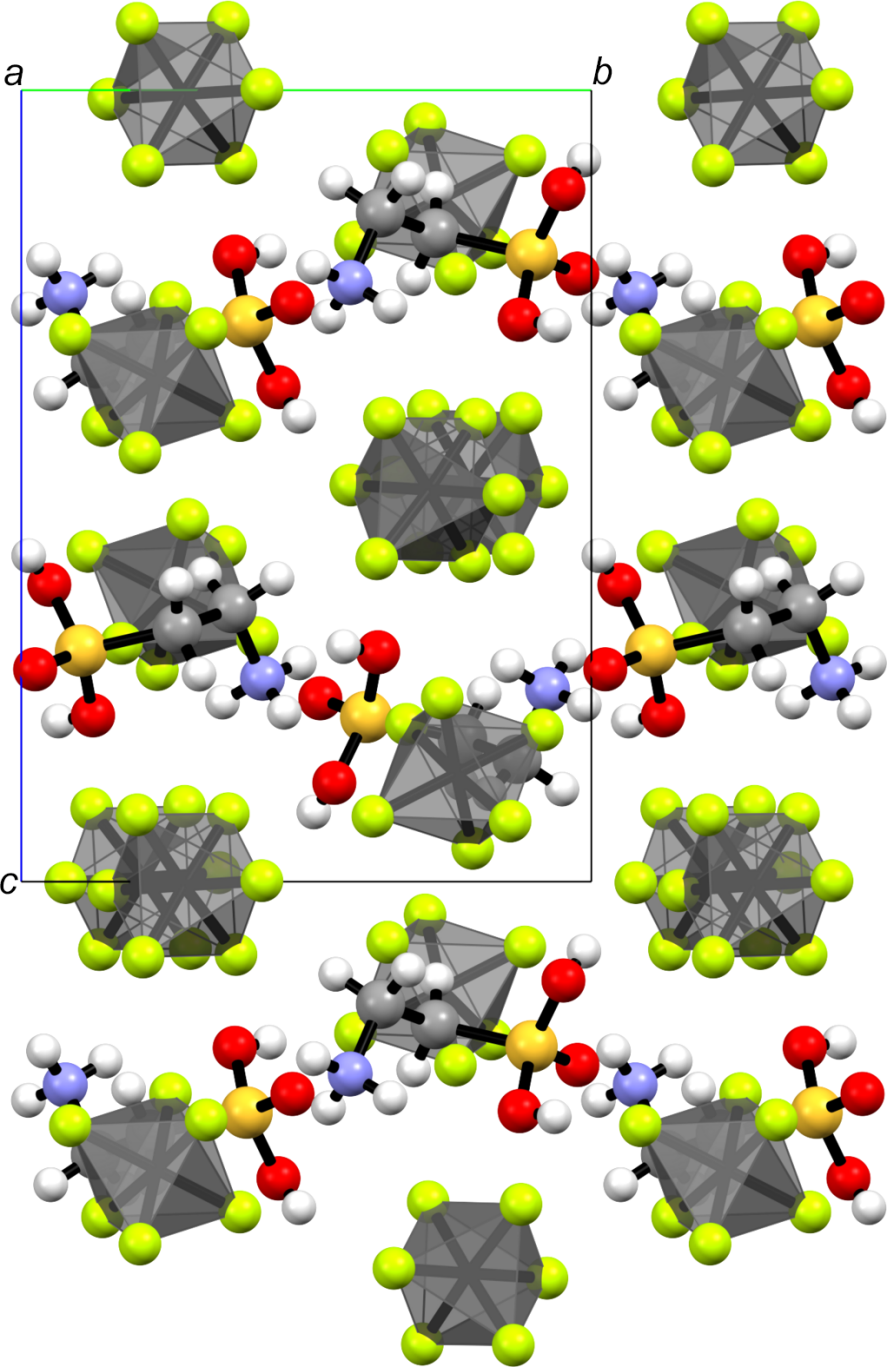
Polyhedral illustration of the slicing in the crystal structure of [H_2_O_3_SC_2_H_4_NH_3_][AsF_6_]_2_, viewed along the *a* axis.

In the crystal structure, the diprotonated taurine is surrounded by eight [AsF_6_]^−^ anions and two cations. The cations are arranged in anti­parallel zigzag chains along the *b* axis (Fig. 4 ▸). Two very strong hy­dro­gen bonds are formed, *i.e.* O1—H1⋯F1 [2.522 (5) Å] and O2—H2⋯F7 [2.607 (5) Å]. Medium–strong hy­dro­gen bonds are found in the range 2.776 (5)–3.161 (6) Å (Table 4 ▸). In accordance with the criteria given by Jeffrey, the assignment of weak/strong hy­dro­gen bonds shows short and directed contacts for strong hy­dro­gen bonds, and longer and nondirectional contacts for weaker hy­dro­gen bonds (Jeffrey, 1997 ▸).

Atom As1 is sourrounded by atoms F1–F6 and As2 by F7–F12, with As—F bond lengths in the range 1.688 (3)–1.759 Å. The [AsF_6_]^−^ octa­hedra are slightly distorted com­pared with idealized *O_h_* symmetry in [AsF_6_]^−^ (Biswal *et al.*, 2012 ▸), through elongation of the As—F bond along the strongest hy­dro­gen bonds in the crystal structure (Table 6 ▸).

### NMR spectroscopy

The ^1^H, ^13^C, ^14^N and ^19^F NMR spectra of taurine were measured in anhydrous hydrogen fluoride (aHF) and in the binary superacidic medium aHF/AsF_5_.

The ^1^H NMR spectrum (see Fig. S3 in the supporting in­for­mation) shows three visible signals, apart from the solvent HF (7.75 ppm) and the external reference acetone (2.05 ppm), *i.e.* a triplet at 5.97 ppm (*t*, C1H_2_, 2H) and two overlapping signals at about 3.42 (sextet, C2H_2_, 2H) and 3.35 ppm (*t*, NH_3_, 3H). Due to the fast proton exchange in HF, the sulfonic acid moiety might not be visible. In the ^13^C NMR spectrum (Fig. S4), C1 (47.81 ppm) and C2 (36.75 ppm) were detected. In the ^14^N NMR spectrum (Fig. S5), the NH_3_^+^ moiety was detected at −352.18 ppm (*q*, NH_3_^+^). In the ^19^F NMR spectrum (Fig. S6), the only observed signal was assigned to the solvent (HF) at −198.21 ppm, therefore no decom­position was ex­pec­ted.

In order to test whether the protonation reaction in the binary superacidic system is tem­per­a­ture dependent, a second sample was prepared with two equivalents of AsF_5_. Spectra were recorded at −50, −25 °C and room tem­per­a­ture. Because of the low solubility of the synthesized com­pound in HF at −50 °C and the fact that the spectra do not differ to that measured at −25 °C, only two sets of spectra are discussed.

Similar to the starting material, the ^1^H spectrum (Fig. S7) shows two singlets at 9.35 (*s*, H[AsF_6_], 1H) and 5.35 ppm (*s*, C1H_2_, 2H), as well as a triplet at 3.39 ppm (*t*, NH_3_, 2H) and a sextet at 3.42 ppm (*m*, C2H_2_, 2H). The data of the ^1^H spectra suffer from bad shimming. In the ^13^C NMR spectrum (Fig. S8), C1 (47.69 ppm) and C2 (34.86 ppm) were detected. No data were obtained from the ^14^N spectrum, which might be caused by a change of symmetry in the NH_3_ group. Besides the solvent at −142.85 ppm, unreacted H[AsF_6_] was detected at −167.67 ppm in the ^19^F NMR spectrum (Fig. S9).

In the ^1^H spectrum (Fig. S10), a smaller singlet of H[AsF_6_] occurs at 9.33 ppm, indicating a further protonation of taurine. In addition, a triplet at 5.43 ppm (*t*, C1H_2_, 2H) and two signals of the CH_2_ and NH_3_^+^ moieties at about 3.46 (*t*, NH_3_, 3H) and 3.10 ppm (sextet, C2H_2_, 2H), respectively, were observed. In the ^13^C NMR spectrum (Fig. S11), C1 (48.31 ppm) and C2 (35.13 ppm) were detected. The ^14^N NMR spectrum (Fig. S12) shows the NH_3_^+^ moiety at −354.94 ppm (*q*, NH_3_, 3H). In the ^19^F NMR spectrum (Fig. S13), the signals were assigned to the solvent at −144.72 ppm and to H[AsF_6_] at −167.95 ppm, due to residues of AsF_5_.

As monitored by NMR spectroscopy, we expect the pro­to­n­ation of taurine not to succeed in anhydrous hy­dro­gen fluoride at room tem­per­a­ture, as no shift can be detected com­pared to the already known spectra of taurine (Lin *et al.*, 1988 ▸). In the binary superacidic system HF/AsF_5_, monoprotonation is observed at low tem­per­a­ture, with diprotonation observed in excess of Lewis acid at room tem­per­a­ture.

## Conclusion

NMR spectroscopic investigations revealed that the protonation reaction in the binary superacidic systems HF/*M*F_5_ (*M* = As, Sb) is apparently tem­per­a­ture dependent. Thus, less acidic systems, such as HF/BF_3_ or HF/GeF_4_, might also be able to mono- or even diprotonate taurine at room tem­per­a­ture.

As diprotonation of taurine already occurs in less acidic systems, the question may be raised whether taurine can be triprotonated in the stronger acidic system HF/SbF_5_ at room tem­per­a­ture or at even higher tem­per­a­tures in excess SbF_5_, supported by the formation of polyanions (*e.g.* [Sb_2_F_11_]^−^, [Sb_3_F_16_]^−^, *etc*.). As no protonation of sul­fo­nium moieties has yet been observed, it is still unclear whether [H_3_O_3_S–*R*]^2+^ moieties might exist. Therefore, investigations of the protonation of alkyl­sulfonic acids, such as methane­sulfonic acid, might give hints, due to a better stabilizing substituent effect, as shown by the mapped electrostatic potential.

## Supplementary Material

Crystal structure: contains datablock(s) I, global. DOI: 10.1107/S2053229624010489/wv3016sup1.cif

Structure factors: contains datablock(s) I. DOI: 10.1107/S2053229624010489/wv3016Isup2.hkl

Diffraction pattern, crystal image and NMR spectra. DOI: 10.1107/S2053229624010489/wv3016sup3.pdf

CCDC reference: 2394818

## Figures and Tables

**Table 1:** Experimental details

Crystal data	
Chemical formula	(C_2_H_9_NO_3_S)[AsF_6_]_2_
*M*_r_	505.00
Crystal system, space group	Orthorhombic, *P*2_1_2_1_2_1_
Temperature (K)	112
*a*, *b*, *c* (Å)	9.7110 (5), 9.7629 (4), 13.5461 (6)
*V* (Å^3^)	1284.27 (10)
*Z*	4
Radiation type	Mo *K*α
μ (mm^−1^)	5.52
Crystal size (mm)	0.45 × 0.10 × 0.06

Data collection	
Diffractometer	Rigaku OD Xcalibur with a Sapphire3 detector
Absorption correction	Multi-scan (*CrysAlis PRO*; Rigaku OD, 2020 ▸)
*T*_min_, *T*_max_	0.281, 1.000
No. of measured, independent and observed [*I* > 2σ(*I*)] reflections	13161, 3910, 3424
*R*_int_	0.044
(sin θ/λ)_max_ (Å^−1^)	0.714

Refinement	
*R*[*F*^2^ > 2σ(*F*^2^)], *wR*(*F*^2^), *S*	0.034, 0.062, 1.01
No. of reflections	3910
No. of parameters	199
H-atom treatment	H atoms treated by a mixture of independent and constrained refinement
Δρ_max_, Δρ_min_ (e Å^−3^)	0.76, −0.61
Absolute structure	Flack *x* determined using 1283 quotients [(*I*^+^) − (*I*^−^)]/[(*I*^+^) + (*I*^−^)] (Parsons *et al.*, 2013 ▸)
Absolute structure parameter	0.005 (7)

Computer programs: *CrysAlis PRO* (Rigaku OD, 2020 ▸), *SHELXT* (Sheldrick, 2015*a* ▸), *SHELXL2019* (Sheldrick, 2015*b* ▸), *ORTEP-3 for Windows* (Farrugia, 2012 ▸) and *PLATON* (Spek, 2020 ▸).

**Table 2:** Vibration assignment (frequencies in cm^−1^) for diprotonated taurine **[see Note 2]**

Raman	IR^*a*^	Calculated IR/Raman^*b*^	Assignment
3325 (1)	shoulder	3326 (121/24)	ν(NH_3_)
3304 (2)	shoulder	3320 (172/22)	ν(NH_3_)
3251 (3)	3252 (*vs*, *br*)	3257 (57/96)	ν(NH_3_)
3051 (6)	3068 (*vs*, *br*)	3038 (9/34)	ν(C2H_2_)
3016 (11)	shoulder	3005 (20/44)	ν(C1H_2_)
3005 (12)	shoulder	2987 (2/104)	ν(C2H_2_)
2960 (18)	shoulder	2957 (15/104)	ν(C1H_2_)
2906 (2)	2852	2886 (2267/162)	ν(O1H) + (O2H)
2789 (3)	shoulder	2818 (2303/108)	ν(O1H) + (O2H)
1611 (7)	1632 (*w*)	1611 (35/3)	δ(NH_3_)
1585 (8)	1587 (*w*)	1607 (40/5)	δ(NH_3_)
1509 (6)	1491 (*w*)	1490 (147/1)	γ(NH_3_)
1461 (15)	1454 (*w*)	1446 (25/5)	δ(C2H_2_)
1404 (6)		1392 (13/4)	δ(C1H_2_)
1394 (11)		1389 (23/1)	ω(C2H_2_)
1361 (9)	shoulder	1351 (109/7)	ν(S=O)
1342 (17)	shoulder	1323 (55/5)	τ(C2H_2_)
1304 (5)	shoulder	1279 (26/5)	ω(C1H_2_)
1236 (10)	1253 (*m*, *br*)	1225 (50/2)	τ(C1H_2_)
1197 (3)	1213 (*m*)	1216 (50/1)	δ(O1H)
1115 (6)	1118 (*w*)	1203 (25/2)	δ(O2H)
1038 (8)	1090 (*w*)	1085 (31/1)	τ(C1H_2_) + ρ(NH_3_)
1008 (4)	1041 (*w*)	1068 (21/1)	τ(C2H_2_) + ρ(NH_3_)
975 (21)	968 (*w*)	976 (92/2)	ν(C—C) + ν(C—N)
928 (8)	919 (*w*)	935 (173/2)	ν(S—O)
877 (8)	867 (*w*)	889 (102/12)	ν(S—O) + ρ(C1H_2_)
		877 (39/1)	ρ(NH_3_) + ρ(C2H_2_) + ρ(C1H_2_)
832 (23)	825 (*w*)	829 (34/2)	τ(NH_3_) + ρ(C2H_2_) + ω(C1H_2_)
		787 (42/2)	ν(C2—N) + ρ(C2H_2_)
		777 (76/2)	ρ(C1H_2_) + δ(O1H1) + δ(O2H2)
		752 (65/0)	δ(O1H1)
671 (26)	675 (*s*)	637 (39/17)	ν(C—S) + ρ(C2H_2_)
626 (12)	615 (*s*)		
610 (8)			
554 (11)	541 (*w*)		
523 (6)	519 (*w*)	514 (6/2)	γ(SO_3_)
485 (18)	463 (*w*)	445 (23/2)	δ(SO_3_)
473 (16)		430 (4/4)	ω(SO_3_)
438 (9)			
403 (9)	390 (*m*)	404 (47/2)	ρ(C1H2)
300 (9)		309 (16/1)	ρ(C1H_2_) + ρ(C2H_2_) + ρ(NH_3_)
282 (6)		291 (32/3)	
267 (6)		244 (9/0)	
247 (6)		224 (2/0)	τ(NH_3_)
		157 (8/0)	ρ(C2H_2_) + ρ(NH_3_)

Notes: (*a*) abbreviations for IR intensities: *v* = very, *s* = strong, *m* = medium and *w* = weak. IR intensities in kJ mol^−1^ and Raman intensities in Å^4^ u^−1^. Experimental Raman activities are relative to a scale of 1 to 100. (*b*) Calculated at the B3LYP/aug-cc-pVTZ level of theory (scaling factor of 0.968), displaying the relative activity of IR and Ra vibrations.

**Table 3:** Vibrational frequencies (cm^−1^) of the [AsF_6_]^−^ anion (*C*_4*v*_)

Raman	IR	Raman (literature)	IR (literature)
726 (27)		730 (30)	
717 (27)	698 (*s*)	709 (10)	700
690 (100)		680 (100)	
589 (9)		587 (11)	
573 (17)		563 (10)	
403 (9)		400 (15)	400
	390 (*m*)	390 (20)	
373 (39)	374 (*w*)	381 (25)	
	365 (*w*)	363 (25)	

**Table 4:** Hydrogen-bond inter­actions (distances are Å) in the crystal structure of [H_2_O_3_SC_2_H_4_NH_3_][AsF_6_]_2_

Contact	Distance	Contact	Distance
O1—H1⋯F1	2.522 (5)	C2—H2*B*⋯F4^iv^	3.075 (6)
O1—H1⋯F6^v^	2.776 (5)	N1—H3*B*⋯F2^iii^	2.853 (5)
O1—H1⋯F12^ii^	2.904 (6)	N1—H3*A*⋯F5^ii^	2.922 (5)
O2—H2⋯F7	2.607 (5)	N1—H3*C*⋯F4^iv^	2.964 (5)
O2—H2⋯F5^ii^	2.847 (5)	N1—H3*C*⋯F7^viii^	3.023 (6)
O3^i^⋯H1*B*—C1	2.972 (6)	N1—H3*C*⋯F9^vii^	3.161 (6)
O3^i^⋯H3*B*—N1	3.240 (6)	N1—H3*B*⋯F10^vii^	3.026 (6)
C1—H1*A*⋯F11^ii^	3.136 (5)	N1—H3*A*⋯O2	2.998 (6)

Symmetry codes: (i) −*x* + 1, *y* − 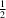, −*z* + 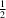; (ii) −*x* + 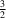, −*y* + 1, *z* − 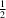; (iii) −*x* + 1, *y* − 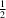, −*z* + 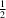; (iv) *x* + 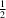, −*y* + 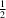, −*z* + 1; (v) *x* + 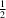, −*y* + 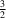, −*z* + 1; (vi) *x*, *y* − 1, *z*; (vii) *x*, *y* − 1, *z*; (viii) −*x* + 2, *y* − 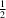, −*z* + 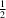.

**Table 5:** Bond-length (Å) com­parison of [H_2_O_3_SC_2_H_4_NH_3_]^2+^ (observed/calculated) with [HO_3_SC_2_H_4_NH_3_]^+^ and [H_2_O_3_SCF_3_]^+^

[H_2_O_3_SC_2_H_4_NH_3_]^2+^	Observed	Calculated			[HO_3_SC_2_H_4_NH_3_]^+^		[H_2_O_3_SCF_3_]^+^			
S1—O1	1.511 (4)	1.562	S1—O1	1.437 (2)	S1—O1	1.505 (2)
S1—O2	1.513 (4)	1.547	S1—O2	1.427 (2)	S1—O2	1.483 (2)
S1—O3	1.410 (4)	1.423	S1—O3	1.548 (2)	S1—O3	1.405 (2)
S1—C1	1.754 (5)	1.801	S1—C1	1.765 (3)	S1—C1	1.855 (2)
C1—C2	1.516 (7)	1.526	C1—C2	1.511 (4)		
C2—N1	1.496 (6)	1.517	C2—N1	1.496 (4)		

**Table 6:** Coordination environment (bond lengths in Å) of [AsF_6_]^−^ units in diprotonated taurine

[As1F_6_]^−^		[As2F_6_]^−^		Na[AsF_6_]	
As1—F1	1.760 (3)	As2—F7	1.750 (3)	As1—F1	1.702
As1—F2	1.718 (3)	As2—F8	1.710 (3)		
As1—F3	1.708 (3)	As2—F9	1.704 (3)		
As1—F4	1.710 (3)	As2—F10	1.688 (3)		
As1—F5	1.711 (3)	As2—F11	1.708 (3)		
As1—F6	1.693 (3)	As2—F12	1.711 (3)		
