# Supplementary material for: Diprotonation of taurine: 2-[dihy­droxy(oxo)sul­fan­ylium­yl]ethanaminium bis[hexa­fluoro­arsenate(V)]
Source: Acta Crystallogr C Struct Chem. 2024 Nov 20;80(Pt 12):781–6. doi: 10.1107/S2053229624010489 (PMC11619780; doi:10.1107/S2053229624010489)
Supplement: Supplementary file 3 [file c-80-00781-sup3.pdf]

Volume 80 (2024)

Supporting information for article:

Diprotonation of taurine: 2-[dihydroxy(oxo)sulfanyliumyl]ethanaminium bis[hexafluoroarsenate(V)]

Valentin Bockmair, Andreas Klöck, Dirk Hollenwäger and Andreas J. Kornath

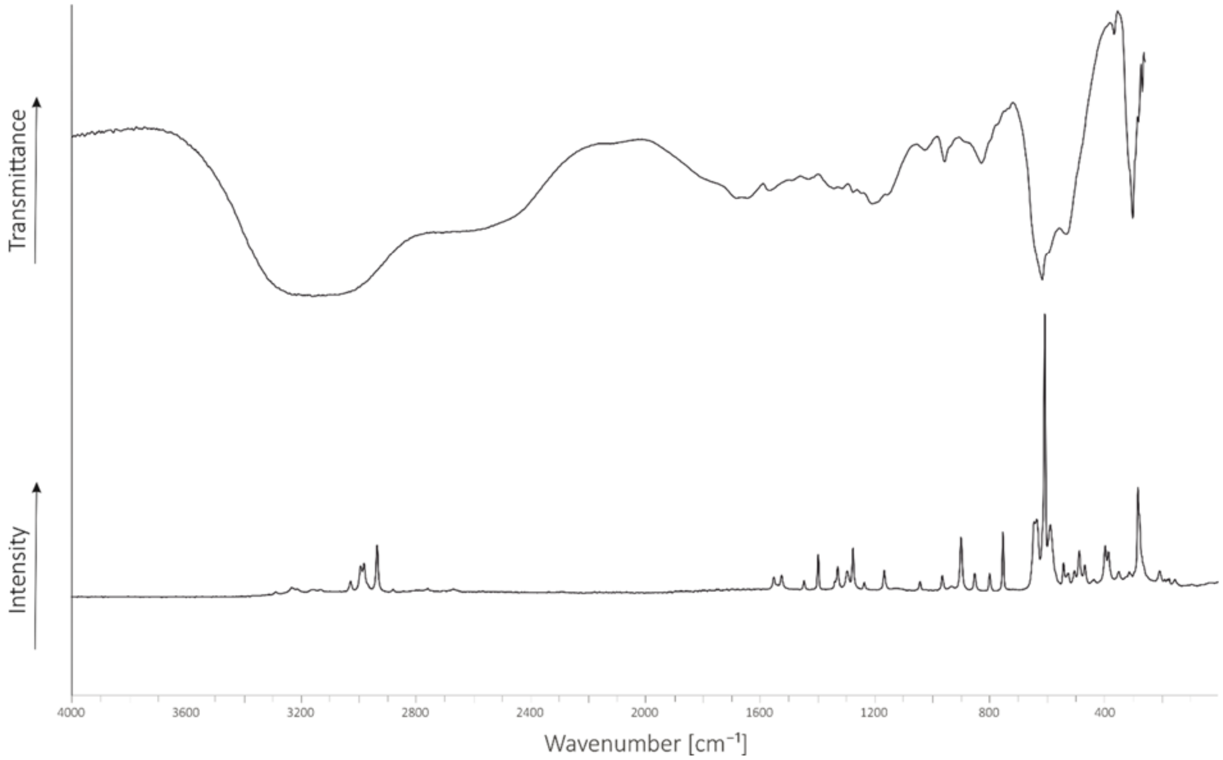

Figure S1 Low-temperature vibration spectra of $[H_2O_3SC_2H_4NH_3][AsF_6]_2$: IR (top) and Raman (below).

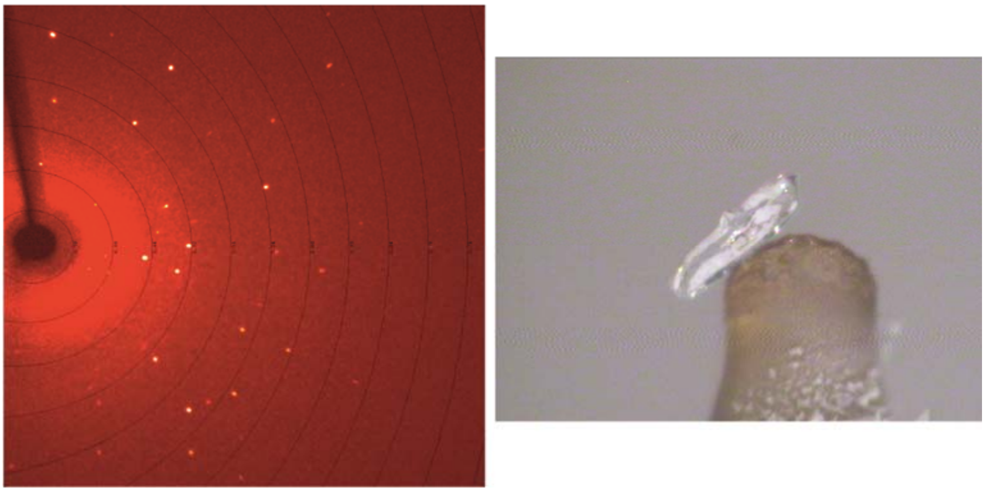

Figure S2 Diffraction pattern (left) and prepared single crystal on polyamide loop of micromount (right).

NMR-spectroscopy

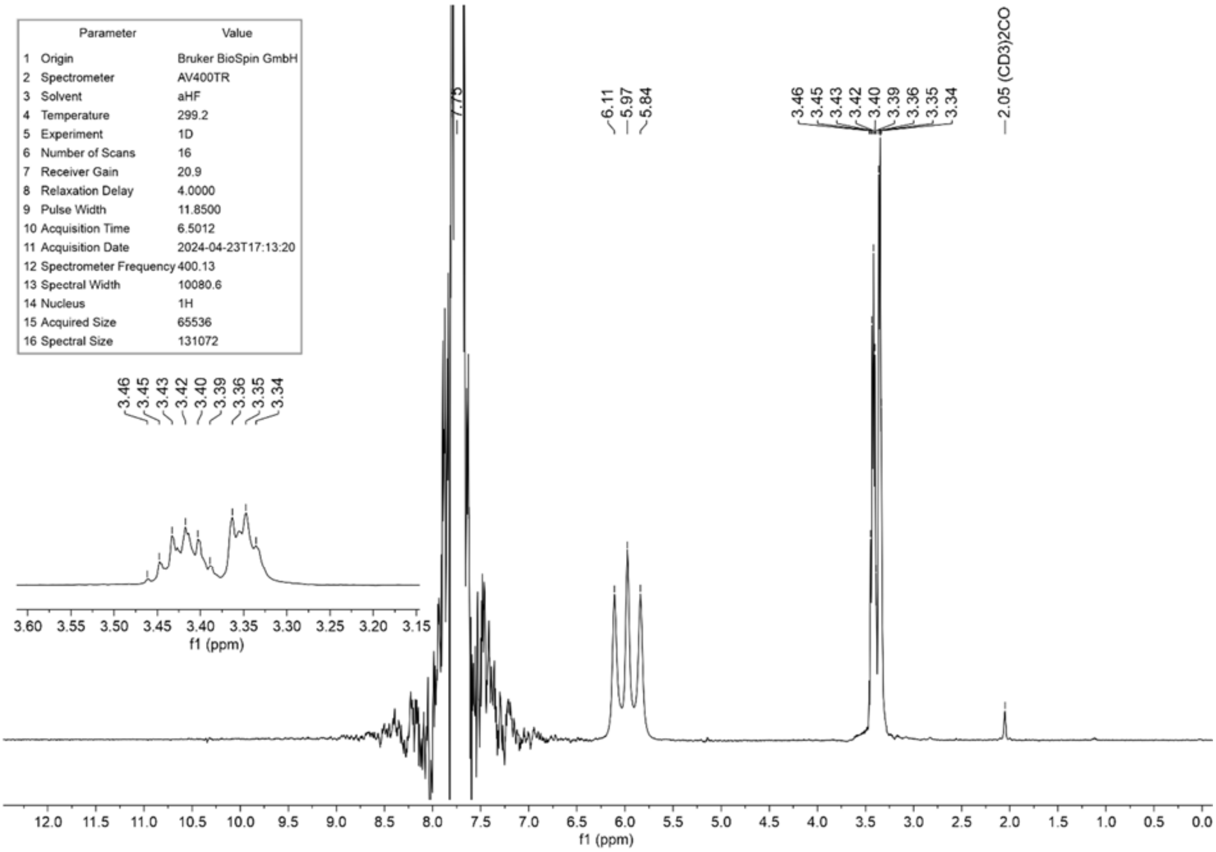

Figure S3. ^{1}H NMR spectrum of taurine in aHF at room temperature.

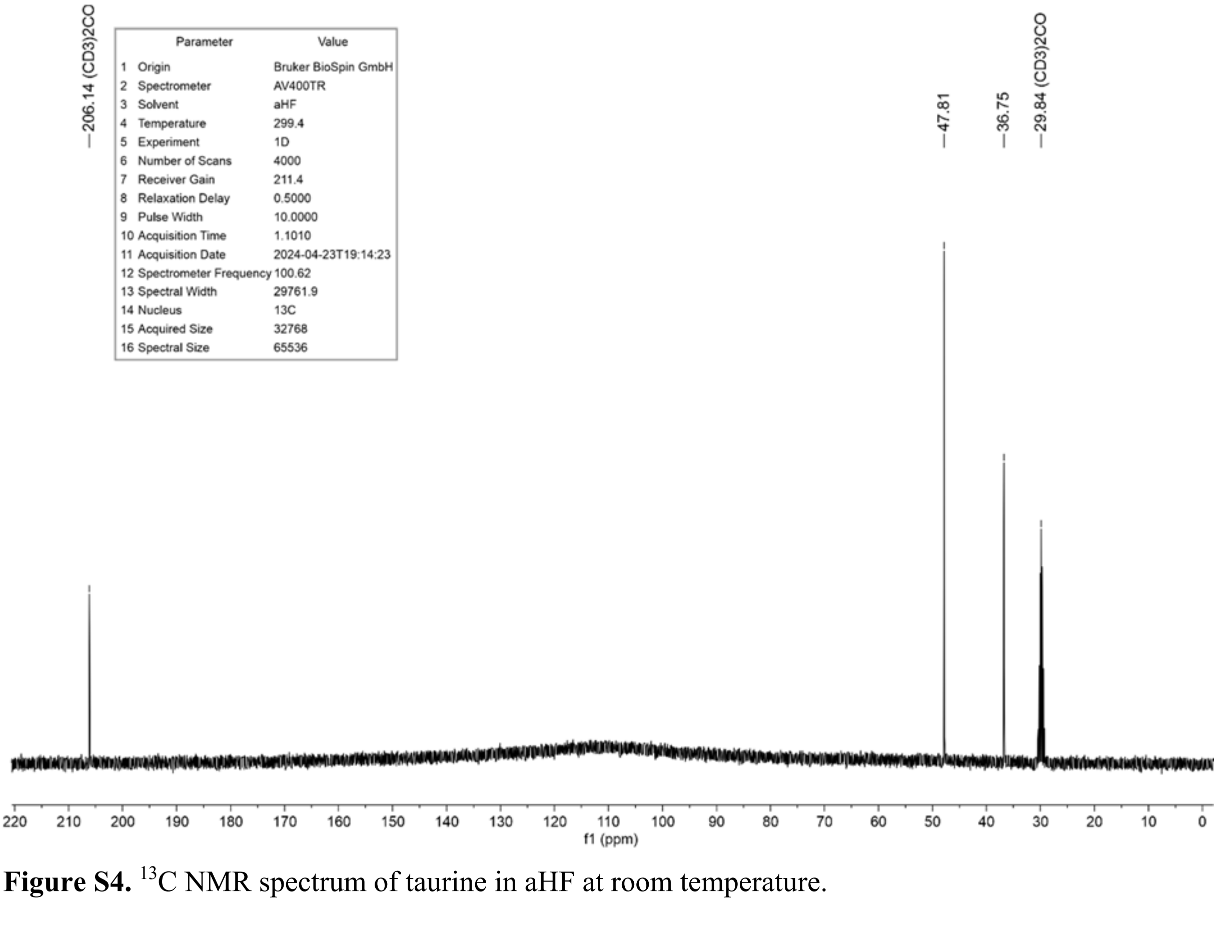

Figure S4. ^{13}C NMR spectrum of taurine in aHF at room temperature.

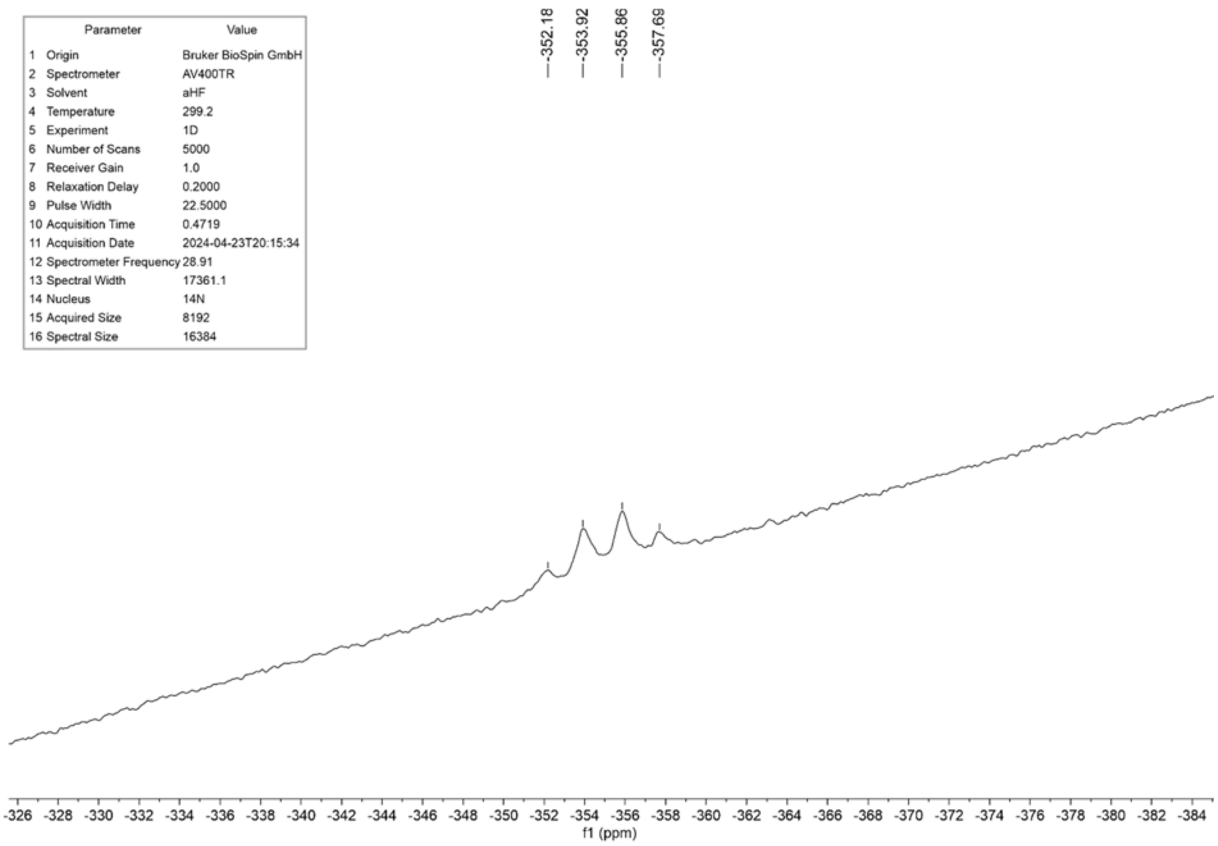

Figure S5. ^{14}N NMR spectrum of taurine in aHF at room temperature.

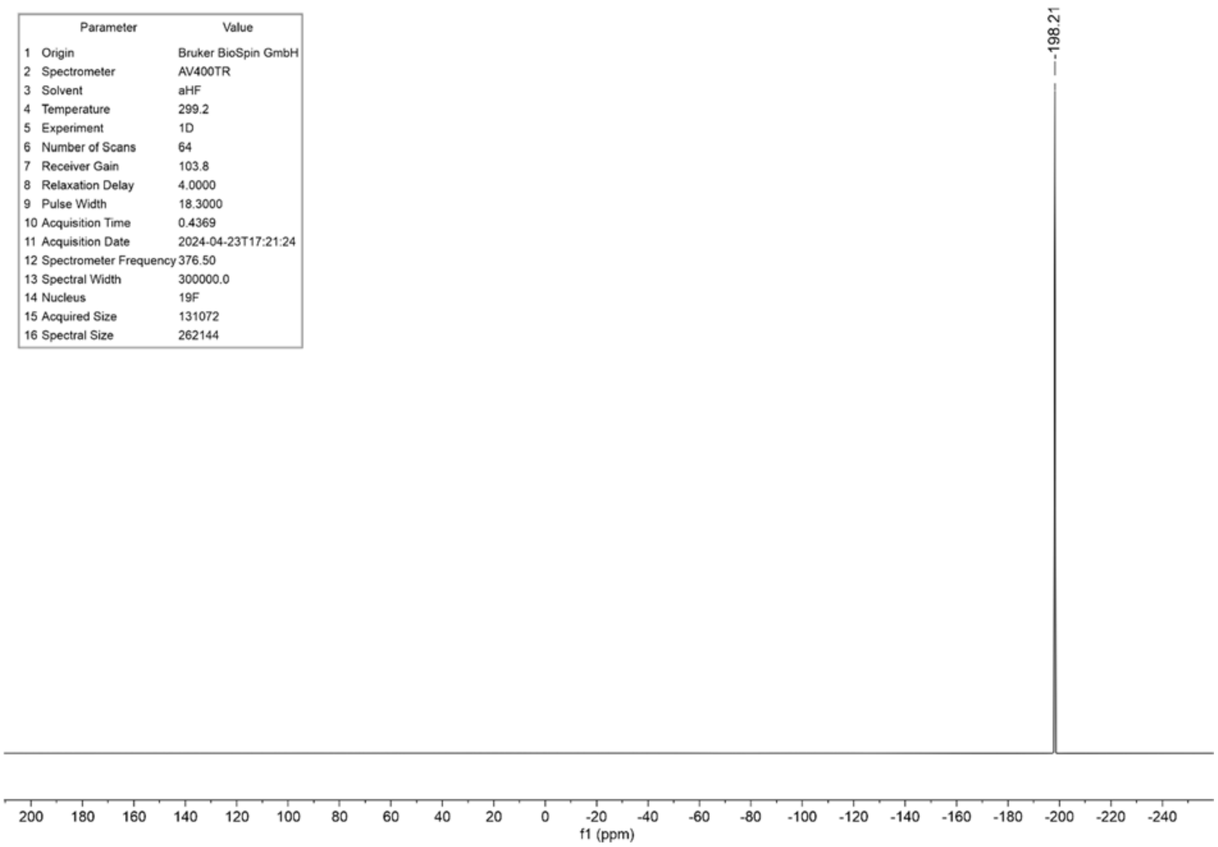

Figure S6. ^{19}F NMR spectrum of taurine in aHF at room temperature.

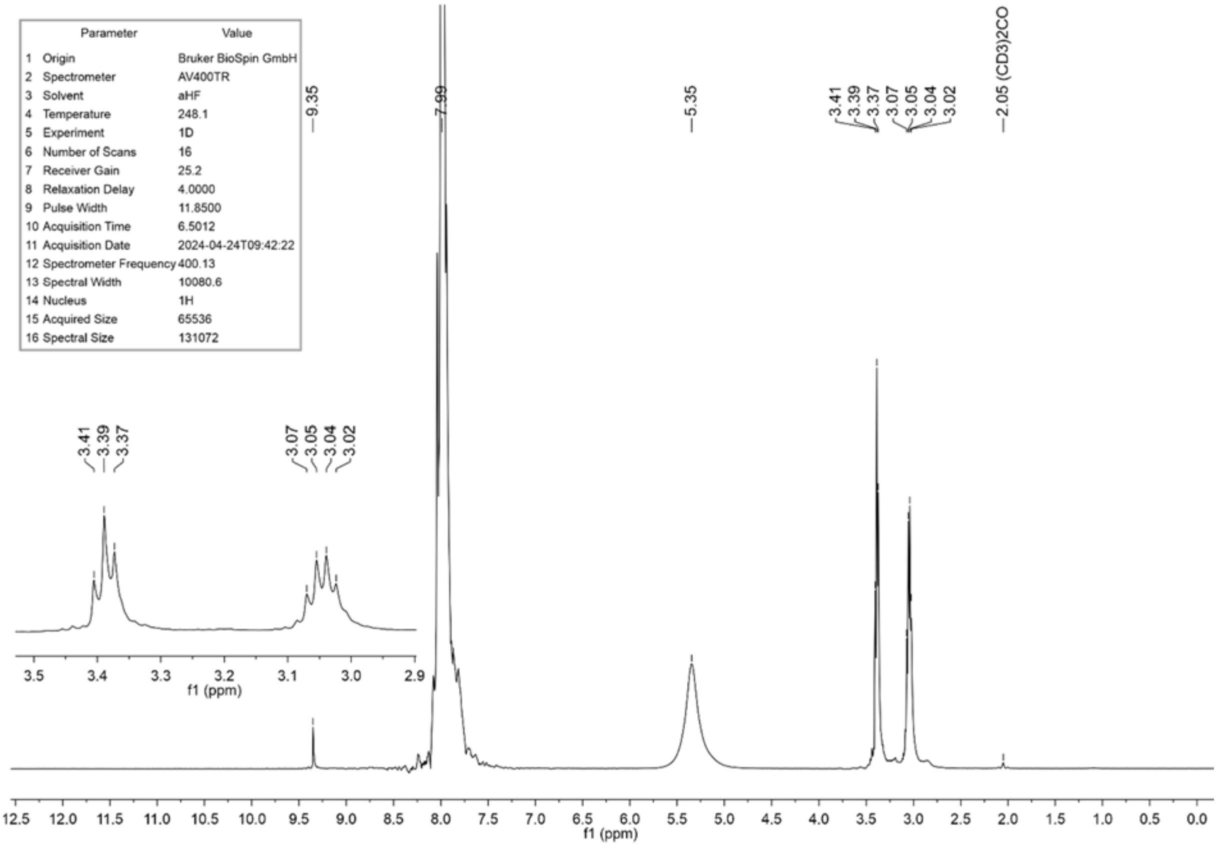

Figure S7. ^{1}H NMR spectrum of taurine in aHF/AsF_5 at –25°C.

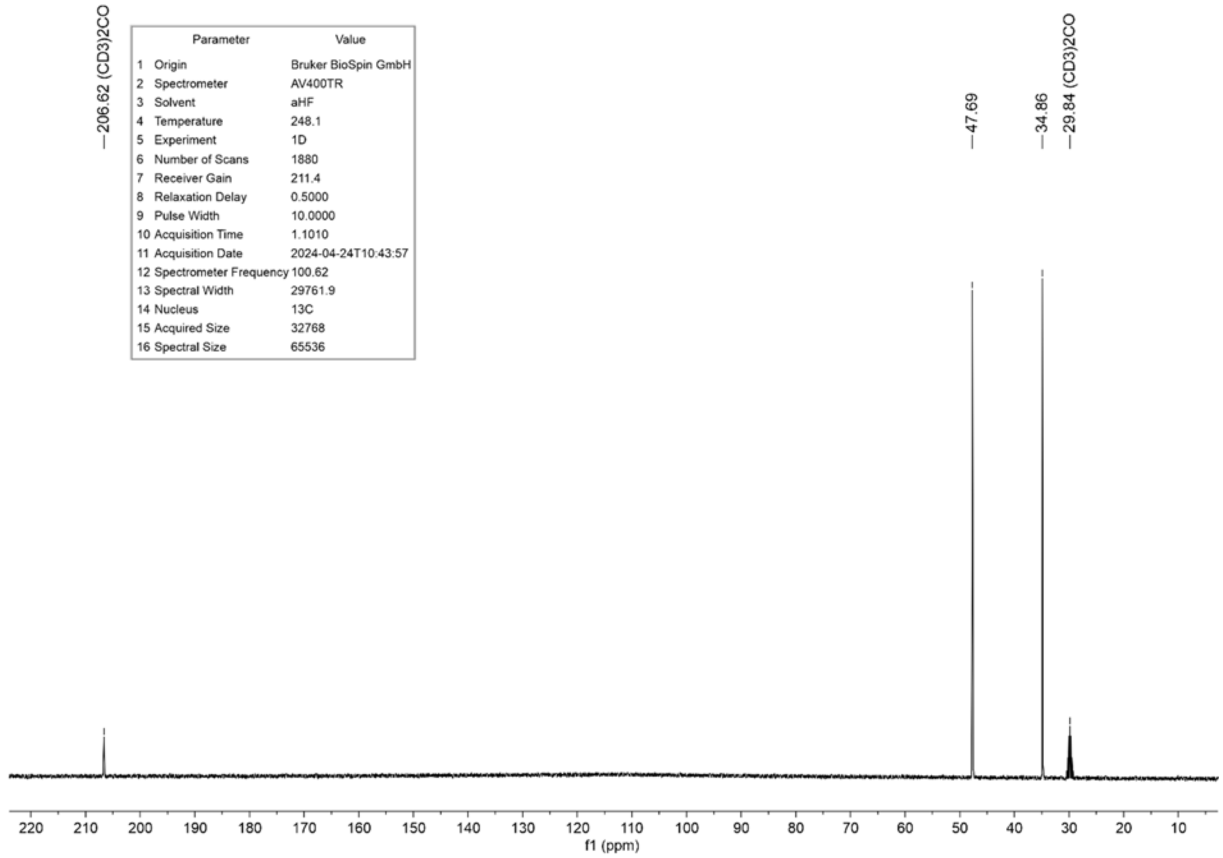

Figure S8. ^{13}C NMR spectrum of taurine in aHF/AsF_5 at –25°C.

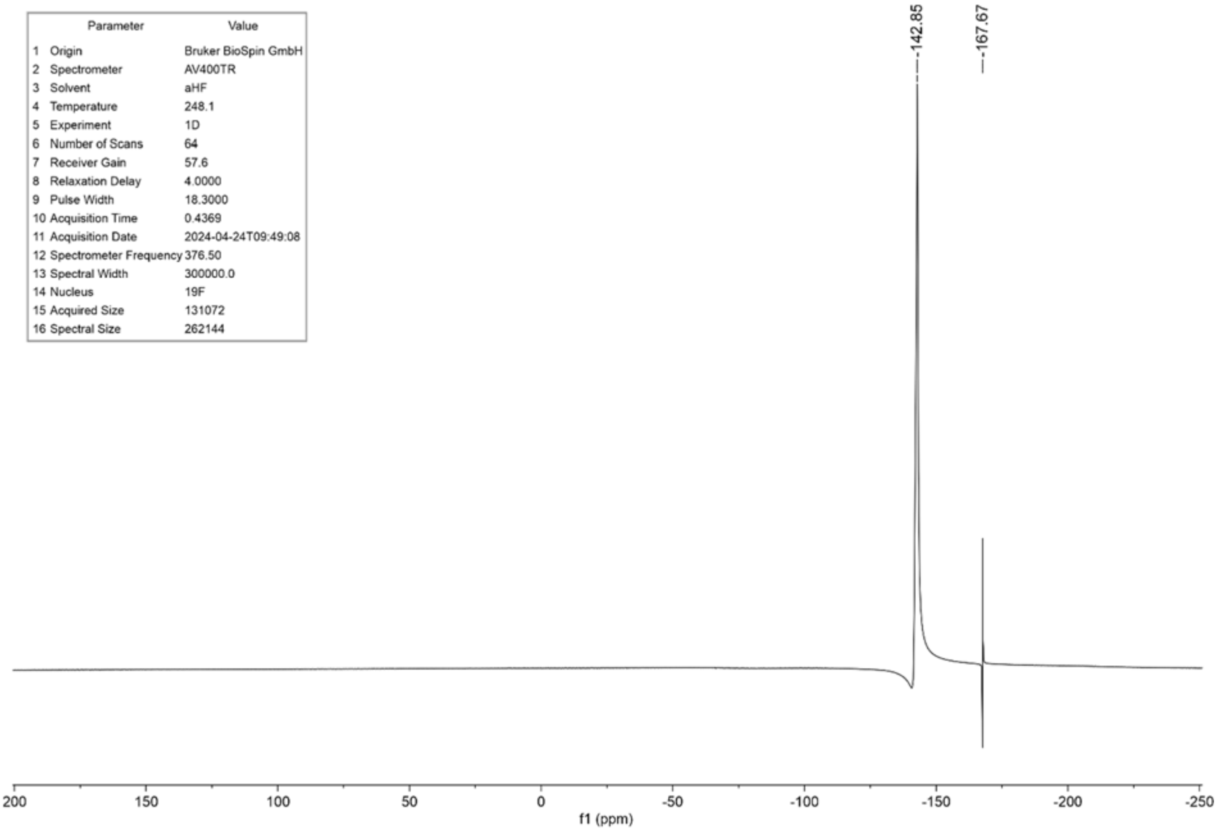

Figure S9. ^{19}F NMR spectrum of taurine in aHF/AsF_5 at –25°C.

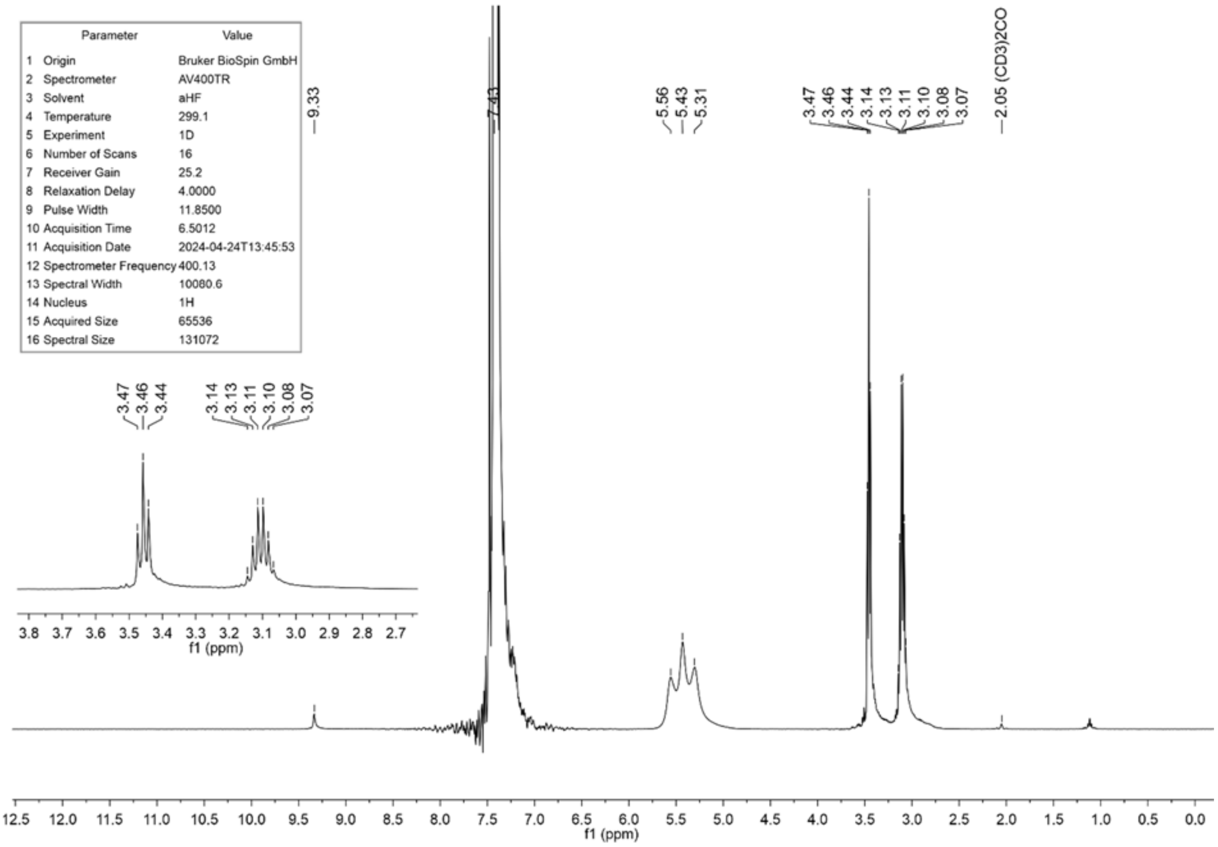

Figure S10. ^{1}H NMR spectrum of taurine in aHF/AsF_5 at room temperature.

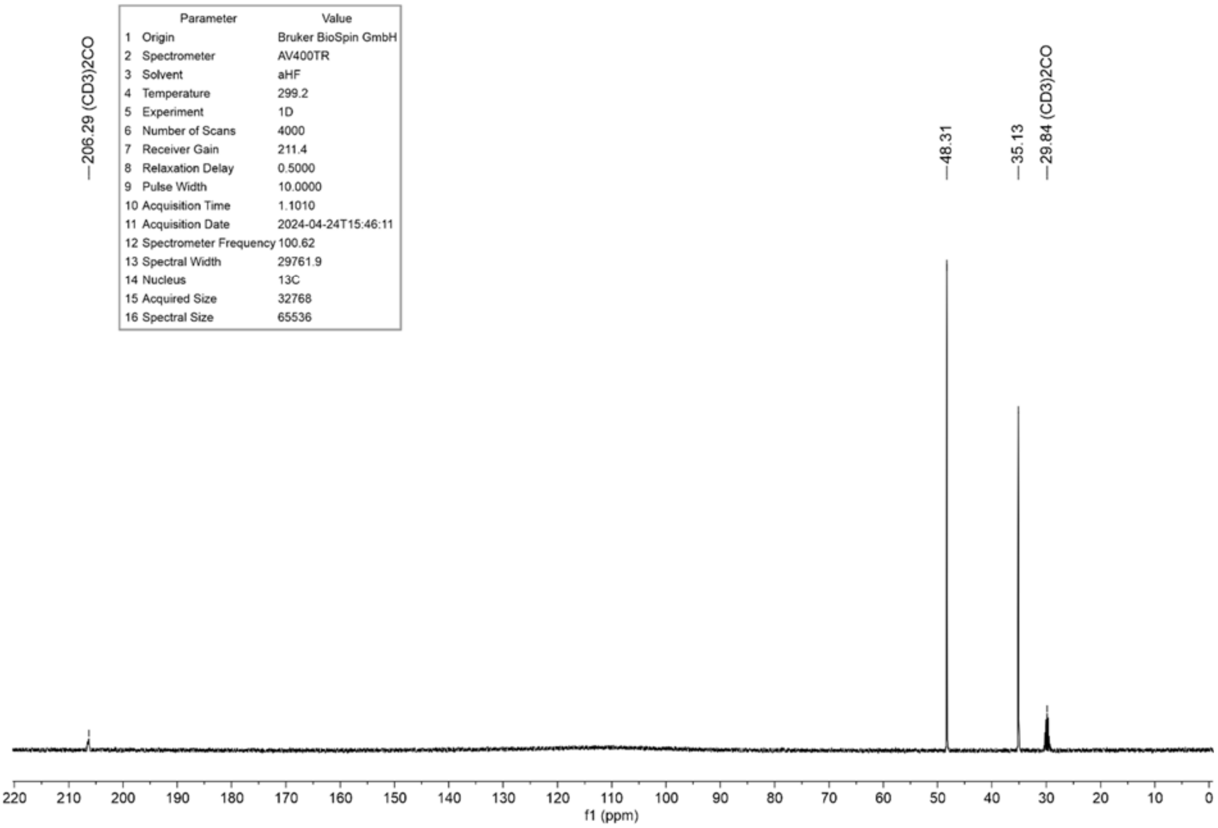

Figure S11. ^{13}C NMR spectrum of taurine in aHF/AsF_5 at room temperature.

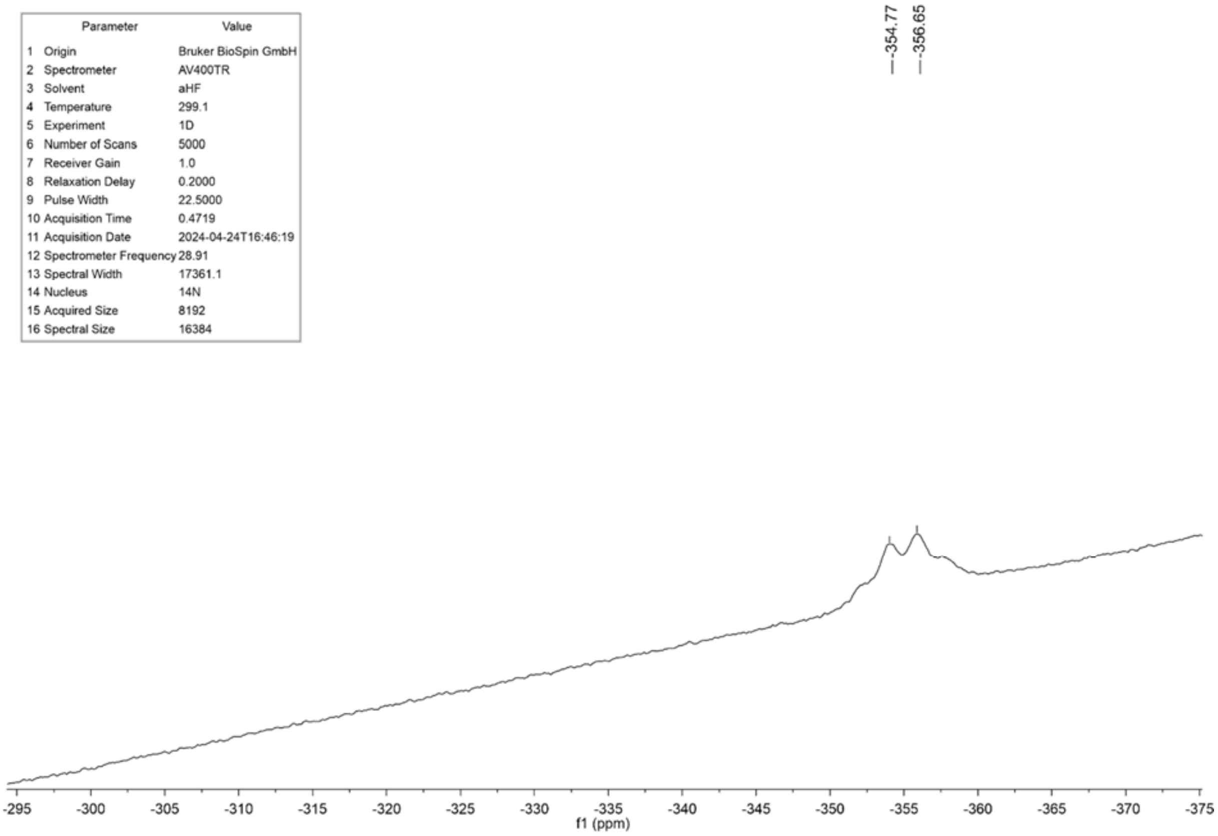

Figure S12. ^{14}N NMR spectrum of taurine in aHF/AsF_5 at room temperature.

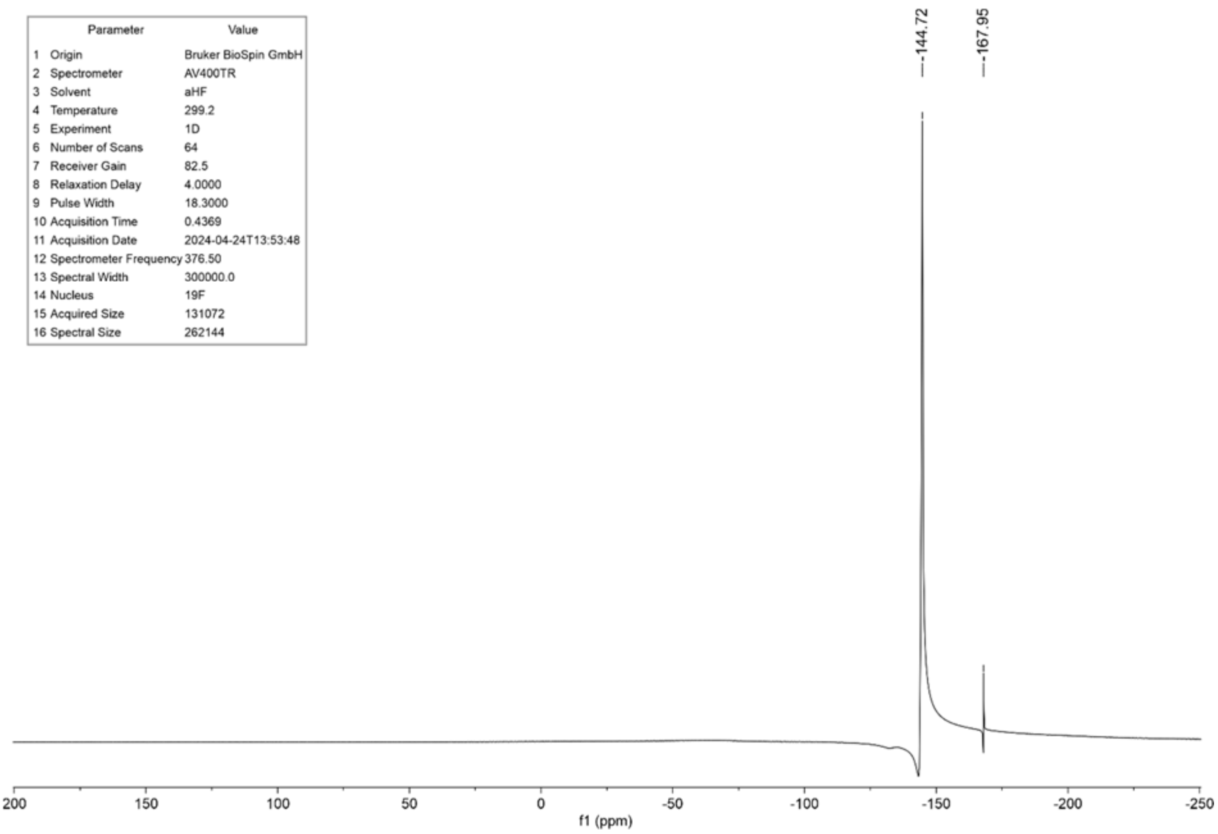

Figure S13. ^{19}F NMR spectrum of taurine in aHF/AsF_5 at room temperature.
